# Influence of an Upper Limb Isometric Task in Perceived and Performance Fatigability of Elderly Subjects: A Quasi-Experimental Study

**DOI:** 10.3390/biology11081175

**Published:** 2022-08-05

**Authors:** Helena Silva-Migueis, Eva María Martínez-Jiménez, Israel Casado-Hernández, Adriano Dias, Ana Júlia Monteiro, Rodrigo B. Martins, Carlos Romero-Morales, Daniel López-López, Juan Gómez-Salgado

**Affiliations:** 1 Research, Health and Podiatry Group, Department of Health Sciences, Faculty of Nursing and Podiatry, Industrial Campus of Ferrol, Universidade da Coruña, 15403 Ferrol, Spain; 2 Physiotherapy Department, Escola Superior de Saúde da Cruz Vermelha Portuguesa—Lisboa, 1300-125 Lisbon, Portugal; 3 Facultad de Enfermería, Fisioterapia y Podología, Universidad Complutense de Madrid, 28040 Madrid, Spain; 4 Epidemiology—Department of Public Health and Grade Program of Public/Collective Health, Botucatu Medical School/UNESP, Botucatu 18618-687, Brazil; 5 Faculty of Sport Sciences, Universidad Europea de Madrid, Villaviciosa de Odón, 28670 Madrid, Spain; 6 Department of Sociology, Social Work and Public Health, Faculty of Labour Sciences, University of Huelva, 21071 Huelva, Spain; 7 Safety and Health Postgraduate Programme, Universidad Espíritu Santo, Guayaquil 092301, Ecuador

**Keywords:** elderly, isometric activity, fatigue, muscle fatigue, quality of life, physical activity

## Abstract

**Simple Summary:**

This study aimed to understand the influence of an upper limb isometric task on fatigability behavior and the role of quality of life and physical activity in the fatigability of elderly participants. It was found that the upper limb isometric task produces changes in perceived and performance fatigability, which are related in the final stage of the activity. Perceived fatigability evolved progressively with a major increase in the second half of the activity. Changes in fatigability were related to BMI and health-related quality of life dimensions. Considering the results of our study, the use of perceived fatigability as a regulatory factor during upper limb isometric tasks, especially in the clinical context, should be carried out with caution. Additionally, the results highlight the need for the implementation of active aging programs that promote functionality, weight control, and social net reinforcement strategies that may reduce fatigability in the elderly.

**Abstract:**

Isometric activity can be used as a strategy to improve health, fitness, and functional performance in the elderly population, but differences in fatigability may occur. This study aimed to understand fatigability behavior during an upper limb isometric task (ULIT) and the role of health status and physical activity in the fatigability of elderly participants. Thirty-two (32) elderly participants (72.5 ± 5.18 years) were instructed to perform ULIT. The Borg CR10 scale and task failure point (TTF) were used to measure perceived and performance fatigability. Self-reported measures were used to assess the quality of life and physical activity level. A significant relationship between perceived and performance fatigability was found only in the final phase of activity (*p* < 0.01). Significant correlations were found between perceived fatigability and the social functioning dimension (*p* < 0.05), and between performance fatigability (TTF) and BMI (*p* < 0.01), physical functioning (*p* < 0.01), and role functioning/physical (*p* < 0.05) dimensions. In conclusion, ULIT produces changes in fatigability of elderly people, which are positively related in the final stage of the activity. Changes in fatigability are negatively related to BMI. It is also negatively related to health, social functioning, physical functioning and role functioning/physical quality of life dimensions.

## 1. Introduction

In healthy individuals, young or older, fatigue is a protective, transient, and predictable symptom that decreases with rest and does not interfere with daily activities. 

However, the prevalence of fatigue is high and increases with advancing age [1,2,3], and is one of the five criteria of frailty status in the elderly (fried phenotype) [4], and commonly, the most disabling symptom (with pain in second place) [5]. Fatigue interferes in the performance of daily tasks and compromises functional performance [1,6] particularly with upper limb tasks [7,8,9,10,11,12], and especially those requiring unsupported arm elevation [13,14]. It can also lead to decreased social interaction [15] and predict later health services usage [16] and mortality [17]. 

The high prevalence of fatigue in the elderly may be caused by underlying medical conditions, aging physiological deterioration (12), decrease in physical activity, even in daily tasks [18], and poorer self-rated health which seems to be related to higher fatigue scores in the general population [19]. 

Fatigue is often described as decreased vitality, loss of energy, anergia, exhaustion, tiredness, weakness, and lassitude [20], but is defined as “*a symptom in which physical and cognitive function is limited by interactions between performance fatigability and perceived fatigability*” [21].

Distinct from fatigue, fatigability puts the fatigue in the perspective of a specific activity, simplifying the comparison across samples with different functional health [22].

Perceived fatigability is the subjective sensation of weariness, increasing sense of effort, and mismatch between effort spent and actual performance, or exhaustion [7,21,23]. It reflects the changes in sensations that regulate the performer’s integrity and depends on the physiological capacity of the body to keep homeostasis, and the psychological state of the individual, where the relative weight of each factor and their reciprocal interaction depends on the conditions or disease [23]. 

Performance fatigability, described as the “*decline in an objective measure of physical performance over a discrete period*” [21], depends on peripheral muscles and central nervous system capacity to fulfill the requirements of a physical task [23] and is measured using a variety of physical tasks [24], where outcome variables can be the duration that a task can be sustained (time to task failure), the rate of change in muscular activation, power production, and other physiological parameters [21]. Task demands, such as contraction intensity and velocity, stability, and support to the fatiguing limb, and the population’s physiological characteristics (sex and aging) determine performance fatigability and the involved mechanisms [25]. 

Fatigability is well validated in older populations and its use is emerging as a tool that complements other functional and behavioral measures for discriminating health and functional status [20,26].

Considering that the elderly population continues to increase [27], health maintenance and physical independence in this population is a global goal [28]. Fatigue and pain are the major symptoms in this population [5], and isometric activity can be an important strategy for recreational, sports, and rehabilitation plans to improve health, fitness, and functional performance, since its benefits include improved joint stability (without joint movement), lower blood pressure [29,30] and decreased overall pain [31].

However, because psychophysiological adjustments and rate-limiting mechanisms occur during fatiguing isometric tasks and they differ between populations, differences in fatigability also arise. This has special relevance to position sustained isometric tasks (with minimal limb support) where load compliance needs for the maintenance of the limb are much greater than for other types of tasks [32]. 

Understanding how fatigability behaves in the elderly can help to understand its role along the disablement pathway and identify risk factors in this population but also develop more targeted practices for the needs, expectations, and behavior of the elderly, increasing their effectiveness and sense of self-efficacy either in sports, occupational or rehabilitation contexts. 

Therefore, the present study aims to understand the influence of an upper limb isometric task on perceived and performance fatigability behavior and the role of quality of life and physical activity in the fatigability of elderly participants. In doing so, we established two hypotheses: (1) upper limb isometric tasks produce highly correlated changes in perceived and performance fatigability, and (2) changes in perceived and performance fatigability are related to demographic and anthropometric characteristics, quality of life, and physical activity of the participants.

For this purpose, perceived fatigability was measured using the Borg CR10 scale and performance fatigue was registered as the time to task failure (TTF). HrQoL dimensions scores were e extracted from SF36 v2 and physical activity levels were extracted from the EPIC-PAQ index to investigate associations with these variables.

## 2. Materials and Methods

### 2.1. Design and Sample

A quasi-experimental prospective study was conducted. The methods and results of this study are reported according to the CONSORT 2010 [33] guidelines.

Through surrounding community institutions, community-dwelling elderly people (≥65 years old) were invited to participate in the investigation, between October 2021 and February 2022. 

Respondents were excluded if they had: (1) a history of heart, cardiovascular, and/or respiratory disease, known untreated hypertension, cardiomyopathy, or exercise intolerance, that can raise the risk of cardiovascular abnormalities during isometric activity; (2) evidence of cognitive or neurological disorders that cause participants to be unable to comprehend or comply with the study procedures; (3) Body Mass Index (BMI) ≥ 40 to prevent the risk of cardiovascular abnormalities associated with class III obesity during physical activity; or (4) neuromuscular or orthopedic disorders that limit the movement of the upper limb to 90° flexion or the maintenance of that position.

**Figure 1:**
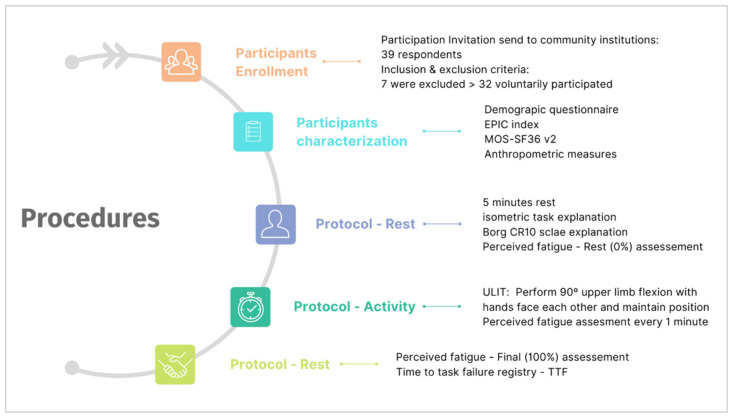
Sampling and protocol process chart.

Of the 39 respondents assessed for eligibility, 7 were excluded due to the criteria (3 for exclusion 1, and 4 for exclusion criteria 4), and 32 voluntarily accepted to participate (16 male and 16 female) (Figure 1) with ages between 65 and 85 years old (age: 72.50 ± 5.18 years; weight: 73.34 ± 12.83 kg; height: 1.59 ± 0.08 m; BMI: 28.72 ± 4.58 kg·m^2^).

### 2.2. Procedure

The study protocol was conducted at CrossLab-Health Research Lab at Escola Superior de Saúde da Cruz Vermelha Portuguesa—Lisboa in Portugal in a temperature-controlled room set to 23 °C.

All the participants followed the same general protocol, and all measurements were taken by the same investigators. The participants were instructed to not drink caffeine beverages for 2 h before the experiment and not to drink alcohol on the day of the experiment. 

First, participants were weighed, measured, and asked to complete a brief characterization questionnaire, the EPIC-PAQ index [23] and MOS-SF36 v.2 [24,25] questionnaires to assess physical activity level and health status. After completing the questionnaires, the participants were asked to sit and stay relaxed for 5 min in a chair with back support with feet resting on the floor. Recommendations were made about the task that they should do and the use of the Borg CR10 scale [26] was explained according to Borg’s recommendations [27]. Blood pressure and rest fatigability were measured, using the Borg CR10 scale.

After five minutes, participants were asked to do the task, which consisted of upper limbs flexion until 90° with hands facing each other and kept in that position (unsupported isometric activity of the upper limbs at 90° flexion from the anatomical position) for as long as they could. The participants reported their intensity of fatigue felt during the activity according to the Borg CR10 scale every time they heard a sound, which was played every 1 min until the end of the task. 

Immediately after task cessation, the participants were asked to assume a resting position and report their final CR10 score as well as the motive for the task cessation. Blood pressure was assessed and total activity time until task failure point (time to task failure point-TTF) was recorded.

### 2.3. Outcome Measures

The activity-related perceived fatigability was assessed through the Portuguese version of the Borg 10 points category-ratio scale (Borg CR10 Scale^®^). The Borg CR10 Scale is a general intensity scale with category-ratio properties better suited to the subjective sensations of exertion, such as local fatigue, breathlessness, dyspnea, discomfort, and pain [34]. For this study, participants were instructed to report perceived exertion for local fatigue or discomfort in the upper limbs.

Time to task failure (TTF) in seconds was used as a performance fatigability outcome.

Health status was measured with the Medical Outcomes Study Short Form Health Survey 36 Item version 2 (MOS-SF-36v2^®^), which is considered a valid and reliable instrument for the Portuguese population [2,3,27,28,35,36]. 

SF-36 is a self-administration questionnaire that can be used by persons 14 years of age and older to measure self-perception of health status across age, disease, and treatment groups. It includes one multi-item scale that assesses eight health concepts: limitations in physical activities because of health problems; limitations in social activities because of physical or emotional problems; limitations in usual role activities because of physical health problems; body pain; general mental health (psychological distress and well-being); limitations in usual role activities because of emotional problems; vitality (energy and fatigue); general health perceptions. The scores range from 0 (worst health status) to 100 (best health status) [37,38]. 

Physical activity level was assessed using the Portuguese version of the European Prospective Investigation into Cancer and Nutrition Physical Activity Questionnaire (EPIC-PAQ) [21]. This questionnaire allows the estimation of energy expenditure through a self-reported assessment of the intensity and average duration of physical activity performed by the participants in three distinct dimensions (occupational, domestic, and leisure). Participants were categorized as “Active” if they reported at least 150 min per week of moderate physical activity, or at least 75 min per week of vigorous physical activity; or at least 150 min per week of a combination of moderate/vigorous activity. If they do not report any of the conditions described, they were categorized as “Sedentary” [39].

### 2.4. Ethical Considerations

The study followed international principles stated in the Declaration of Helsinki [40]. Was approved on March 5, 2021, by the Escola Superior de Saúde da Cruz Vermelha Portuguesa—Lisboa Ethics Committee, Portugal (ESSCVP-EC_01/2021) and was prospectively registered at ClinicalTrials.gov (NCT04938791). Before beginning the study, each subject was informed as to the purpose and content of the investigation. The informed consent was read by the volunteer participants and all gave their written informed consent.

### 2.5. Sample Size Calculation

We used the software G * Power 3.1.9.2 (G * Power ©; University of Düsseldorf; Germany) to calculate the sample size to observe differences before and after the intervention study with a statistical confidence of 95%. Therefore, a 2-tailed hypothesis test and a large effect size of 0.90, an α-error of 0.05, and a power of analysis of 0.80 (β error = 20%) were selected. The result obtained was 18 participants. Considering the possibility of loss to follow-up, a total of 39 participants were recruited.

### 2.6. Statistical Analysis

Due to the heterogeneity of participants’ activity duration, and so, the number of measures of the perceived fatigability, it was necessary to perform normalization of perceived fatigability scores, to make them comparable. Thus, five stages were established corresponding to perceived fatigability (PcFat) in a certain percentage of activity time (0, 25, 50, 75, and 100 % of TTF). These normalization stages have previously been used [41], and in our study, were established by attending the minimum value of TTF (128 s) and ensuring a minimum of 30 s between cut-offs [42].

Variation of perceived fatigability (VPcFat) between two stages was also calculated.

The demographic and anthropometric characteristics and variables of the participants were summarized as mean, standard deviation (SD), and maximum and minimum values. Categorical variables, such as sex, BMI class, and physical activity class were presented as frequency and percentage values.

The Shapiro–Wilk test was used to investigate the variables’ normal distribution (*p* > 0.05). For the analyses of normal variables, parametric tests were used: Student’s *t*-test for independent samples to determine the existence of significant differences between means of sexes and physical activity level groups; Student’s *t*-test for paired samples to determine significant differences between means of two moments; Levene’s test to check the equality of variances; and Pearson correlation coefficient to measure the strength of a linear association between variables [43]. For non-normal distributed variables, non-parametric tests were used: the Mann-Whitney U test for independent samples to compare variables differences between sexes and physical activity level groups; the Kruskal-Wallis test for independent variables to compare BMI class groups; the Wilcoxon signed-rank test to compare differences in fatigability variables in different moments; and Spearman’s correlation coefficient to measures the strength and direction of association between variables. In all the analyses, a *p*-value < 0.05 with a CI of 95% was considered statistically significant. The effect sizes were analyzed and confidence intervals of the *r* values calculated [44]. The statistical analyses were performed using SPSS statistical software, version 25.0 for Windows (IBM Company, Armonk, NY, USA).

## 3. Results

The results of the Shapiro–Wilk test demonstrated that only some variables were normally distributed (*p* > 0.05), namely: age, weight, height, BMI, MOS-SF36 physical functioning, vitality, general health, perceived fatigability at 25% (PcFat 25%) and 50% of TTF (PcFat 50%), variation of perceived fatigability between 25% and 50% of TTF (VPcFat 25–50%), between 0% and 50% of TTF (VPcFat 0–50%), between 50% and 100% of TTF (VPcFat 50–100%) and between 25% and 75% of TTF (VPcFat 25–75%).

### 3.1. Sample Demographic, Anthropometric, Physical Activity, and Clinical Characteristics

A total sample of 32 participants (16 men and 16 women) completed the investigation. The participants were aged between 65 and 85 years (mean 72.50 ± 5.18 years) and had a mean body mass index (BMI) of 28.72 kg/m^2^ (±4.58kg/m^2^), with 75% of the participants classified as pre-obese or obese [31]. Women and men did not show significant differences in age and BMI, although significant differences were detected in weight and height. The participants’ demographic and anthropometric characteristics are presented in Table 1.

**Figure 2:**
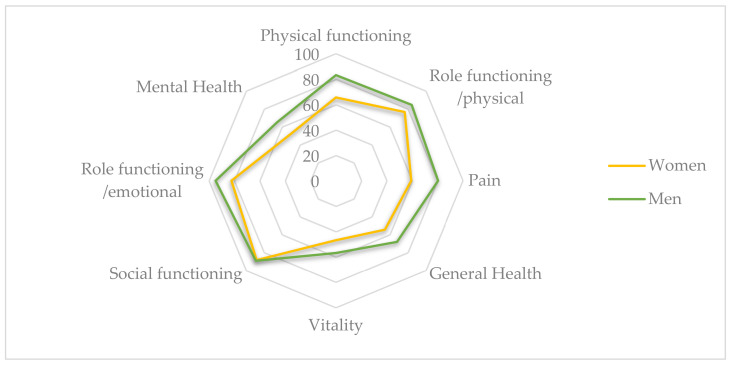
MOS-SF36 health-related quality of life dimensions: women vs. men.

Social functioning (88.67 ± 15.99) and role functioning/emotional (88.54 ± 18.54) were the two health dimensions with highest mean scores, and vitality (51.88 ± 15.75) and mental health (58.25 ± 17.97) had the worst mean scores in SF36 (Figure 2). Men showed higher mean scores in all health dimensions, but statistically significant differences related to sex were identified in physical functioning (*p* = 0.009), pain (*p* = 0.023), and mental health (*p* = 0.039) dimensions, with men having higher mean scores than women (Table 2.).

The mean physical activity time spent in moderate to vigorous activities was 646.53 min per week (±701.66 min/week) and about 69% (22 participants) were considered active. Women spent significantly (*p* = 0.035) more time performing moderate-intensity activities (696 ± 180 min/week) than men (263 ± 75 min/week). Men dedicate more time to vigorous activities (361 ± 155 min/week) than women (107 ± 72 min/week), although the difference is not statistically significant (Table 3).

### 3.2. Perceived Fatigability and Performance Variables Analysis

Perceived fatigability (PcFat) increased progressively along activity time with significant differences between all analyzed stages (*p* < 0.05), as can be seen in Table 4.

PcFat ranged from 0 to 10 along with the activity, with baseline (PcFat0) and final values (PcFat100) ranging from 3 to 10. No significant differences between women and men were found, although women presented lower median scores (Table 4).

The higher median variation of perceived fatigability scores (VPcFat) was detected in the third and fourth quartiles of activity time: 2.44 points between 50% and 75% of TTF and 2.14 points between 75% and 100% of TTF, resulting in significant differences between the second (25–50% of TTF) and third (50–75% of TTF) quartiles of activity and major variation (4.58 points) of perceived fatigability in the second half of the activity (Table 4).

Regarding performance fatigability, the mean TTF was 472.25 s, with men having a higher mean time (572.88 ± 333.94) than women (371.63 ± 221.18 s). A significant negative correlation between TTF and BMI (ρ = −0.471, *p* < 0.01) was found.

The correlation analysis is summarized in Table 5, Table 6 and Table 7. As shown in Table 5 there is a statistically significant relationship between perceived and performance variables, specifically a positive and moderate relationship between PcFat75 and TTF (ρ = −0.544, *p* < 0.01) and PcFat100 and TTF (ρ = −0.645, *p* < 0.01). The sex group analyses show that women follow this tendency, but in men, TTF is related to PcFat25 (ρ = 0.568, *p* < 0.05) and with PcFat75 (ρ = 0.522, *p* < 0.05). 

In the total sample, perceived fatigability is negatively related to the social functioning dimension (Table 6), with significant weak correlations with perceived fatigability at 75% of TTF (ρ = −0.411, *p* < 0.01). However, the relationship between perceived fatigability and quality of life dimensions are different in men and women, with women showing moderate relation only in vitality dimensions and men showing moderate relationships with pain, and vitality.

Despite no significant relation being identified between physical activity total time or physical therapy groups (active or sedentary) and fatigability variables, weak but significant correlations were found between time spent in vigorous activities and perceived fatigability in the end phase of activity (75% of TTF).

No statistically significant correlation was found between BMI and perceived fatigability for the total sample, but in women, age has a positive relationship with PcFat in the initial stages of activity and BMI in the final score of PcFat.

As it can be seen in Table 7, performance fatigability (TTF) appears to be positively and significatively related to physical functioning (ρ = 0.47, *p* < 0.01) and role functioning/physical (ρ = 0.381, *p* < 0.05), but in the group analysis, only women had a statistically significative relation between these variables and physical functioning (ρ = 0.506, *p* < 0.05).

There is also a significant but negative relationship between BMI and TTF, which is also found only in women.

## 4. Discussion

Fatigability is becoming a trend in measuring functional declines, clinical outcomes, and quality of life indicators [44], but to the best of our knowledge, the existing literature associated with fatigability in sustained isometric activities of the upper limbs in older people is very scarce and no study was found that explores perceived fatigability behavior during the execution of the upper limb isometric task and its relationship with performance fatigability.

This study aimed to understand the influence of an upper limb isometric task on perceived and performance fatigability behavior and the role of quality of life and physical activity in the fatigability of elderly participants. Therefore, we found that our two hypotheses that were both partially confirmed since in a sample of elderly participants, (1) ULIT produced a progressive increase in perceived fatigability with intensification in the second half of the activity with related changes in perceived and performance fatigability only in the final phase of activity, and (2) changes in perceived and performance fatigability were related to anthropometric characteristics (BMI), and health-related quality of life dimensions, but not with physical activity.

Human activity requires significant interactions between perceived and performance fatigability and its modulators. Homeostatic factors that contribute to perceived fatigability can influence the capacity to generate voluntary muscle activation, which is a factor that influences performance fatigability. Likewise, afferent feedback influences the adjustments required to maintain homeostasis and thus produces modifications to perceived fatigability [7,21].

The interrelation between the two domains is also explored in a study that examined the influence of perceived fatigue in subsequent endurance physical activity [45]. The authors concluded that the perception of fatigue represents a key factor in the regulation of physical performance and is related to metacognitive processes associated with the representation of internal states, the interoceptive awareness, which proved to be a predictor of perceived fatigue. These authors also stated that perceived fatigue may be directly involved in the central regulation of performance, which together with effort and affection, forms part of the sensory experience that signals the activity as (physically and/or psychologically) unattractive. Therefore, like self-efficacy, perceived fatigue may reflect a cognitive factor related to the belief about one’s ability that shapes the amount of effort invested in the task and influences physical tolerance. 

This study also showed that men performed the isometric activity longer than women but with a tendency to higher scores of perceived fatigability and that the relationship between perceived and performance fatigability is stronger in women. However, in men, we found a relationship between the duration of the task (i.e., TTF) and perceived fatigability also in the initial phase of the task (25% TTF). 

Although it is known that sex influences fatigability related to anatomical and physiological differences, with men being usually more fatigable than women for sustained or intermittent isometric contractions, it is also known that these differences are task-specific [41,46], so this hypothesis needs further exploration in studies with other tasks and a larger sample.

Demographic and anthropometric analysis revealed that BMI has a deleterious effect on fatigability (perceived and performance), especially in women, in whom, age also affects perceived fatigability. 

This result confirmed that obesity is a risk factor for higher fatigability with aging, especially in women. In fact, according to earlier research, the probability of having higher fatigability almost doubles for obese people more than 40 years old compared with normal-weight people [47], particularly in shoulder and trunk muscles during an isometric activity at lower work intensities [48]. 

The fatigability increase in obese people may be related to modifications in muscle fiber type composition with the increase in the proportion of fast-twitch fibers (less fatigue resistant than slow-twitch fibers) [48]. This results from an inflammatory process on the central nervous system caused by circulating cytokines produced and secreted by adipose tissue, causing changes in neuronal function and resulting in higher levels of fatigability [49]. This process also leads to an imbalance between energy availability and task demand, as the energetic demands of a physical task increase with BMI, especially when it is caused by an increase in fat mass [49].

As expected, relationships were found between health status dimensions and fatigability behavior, reinforcing the need to expand active and multidisciplinary aging programs, with the promotion of social support, physical and functional activities, and energy regulation strategies in order to enhance the quality of life of this population.

According to the person-centered conceptual model of perceived fatigability [44], variability in participants’ fatigability may be attributed to fairly stable and variable moderating factors such as disease severity, comorbidities, physical fitness, medication regimens, sleep disorders and sleep quality, age, personality, interest/motivation, emotional state, diet, weather, fluctuations in other symptoms such as pain, environmental context, social context, and acute illness [44]. 

Some of these moderating factors are included in quality-of-life dimensions, but in this sample, only social functioning, vitality, and pain revealed a relationship with perceived fatigability, with social functioning being the quality-of-life dimension with a greater impact in the total sample. Vitality and pain showed stronger relationships in sex groups (vitality in women and pain and vitality in men). 

Our results are aligned with the fatigability concept described by other authors [7,21,23], which states that perceived fatigability is theoretically modulated by some homeostatic and psychosocial factors, and performance fatigability is dependent on peripheral and central factors. Social support (i.e., assistance in executing daily activities, namely in transportation, home care, and personal care was identified as a supporting factor against fatigue [50], and in a sample of men, marital and living status modified the association between household activity and perceived fatigability [22]. Another study also showed that the ability of a sample of patients with multiple sclerosis to take part in social roles and activities was strongly and independently associated with fatigue [51]. 

In the face of the aging of the population and possible deterioration of social functioning due to changes in social support, we can expect that there will be direct repercussions on the level of fatigue reported by the elderly in the performance of their daily activities and social participation and consequently on their functional performance and quality of life. 

The dimensions with the greatest impact on performance were the physical functioning and role functioning/physical dimensions. Performance fatigability depends on the capacity of the nervous system to provide adequate activation as well as the muscle’s contractile function [21], and since it produces an acute decline in motor performance, may interfere directly with the ability to perform a variety of activities such as sports, climbing stairs, carrying groceries and walking (i.e., physical functioning) and, consequently, with the performance of normal daily activities such as work, housework or school. However, performance fatigability and the involved mechanisms are task, muscle group, and population-specific, which means that the extent to which performance fatigability affects the quality of life may differ accordingly to people’s sex, age, and tasks performed [25]. 

In addition, the ability to produce and utilize energy changes with aging with a decline in the VO2 peak and an increase in the energetic demands for daily tasks. This contributes to the development of an energy deficit associated with aging whose mechanisms are associated with metabolism, biomechanics, and neuromotor control modifications, which reduce energy capacity and efficiency of movement and increase the mobility costs in the elderly [52,53].

Additionally, in our study, physical activity total time was not associated with fatigability. These results are not in accordance with other studies that show an association between physical activity and fatigability, which is not, however, related to upper limb isometric activity [22,52,54]. Higher physical activity is associated with better cardiorespiratory fitness, and more efficient energy utilization [22]. 

Our results may be related to the fact that physical activity was self-reported, and its values may have been overestimated by participants. Furthermore, physical activity levels precede changes in perceived fatigability [22]. As our sample is composed mostly of active participants, the relationship between the two variables may not yet be significant.

A weak positive relationship was found between the time of vigorous activity and perceived fatigability at the end of the activity, which is reflected only in men. It seems that the time of vigorous activity has an augmentation effect on the perception of fatigue, especially in men, which is the type of activity most performed by them. In their daily routines, women perform significantly less vigorous activity and tend to have lower scores of perceived fatigability, but also a lower time of activity. Therefore, it seems that although vigorous-intensity activities may potentiate higher scores of perceived fatigability, no influence was found in the performance of isometric activities.

The results of this study provide information about fatigue behavior during isometric activity performance in a sample of elderly people that can be applied to develop more secure and targeted practices for the needs and expectations of this population, highlighting the relationships between fatigue and some demographic, anthropometric, physical activity and quality of life variables. 

However, these findings need to be interpreted in the context of some limitations that should be acknowledged. First, a randomization sampling process with a larger sample is required to reduce sample bias. Second, since fatigue is described in multiple ways, fatigue may not be interpreted in the same way by all participants. We attempted to minimize this bias by providing instructions relating to the use of the CR10 Borg Scale and clarifying what we mean by ‘fatigue’ (i.e., global or local weariness and increasing sense of effort); however, this fact may interfere in fatigue intensity classification. Third, physical activity was assessed through self-reporting and may be subject to recall bias. We tried to control this bias, giving examples of tasks that correspond to different intensities of physical activity, but it was not possible to verify the reported median time spent per week. Finally, because the muscle contractions may alter depending on the verbal instructions [29], the type and amount of feedback during task execution may have influenced the study variables. To minimize this bias, feedback was used only to correct limb or trunk position. These limitations should be addressed in future studies.

This study has also some strengths. Our sample included both men and women, providing the opportunity to explore the influence of sex on the results. We examined fatigability as a comprehensive construct that is influenced by biological, psychological, social, and environmental contributors and influences the person as a whole. The use of the HrQoL measure provided the possibility to contextualize fatigability concerning dimensions that influence the quality of life. The use of the EPIC-PAQ index allowed the measurement of physical activity performed in different contexts (occupational, leisure, and domestic) and by type of activity (light, moderate or vigorous intensity). 

In elderly people, timely detection of high fatigability status is essential, and it is necessary to develop targeted measurements and practices that can respond to the needs, expectations, and behavior of this population. This can delay the appearance of fatigue and frailty and increase their functional performance and sense of self-efficacy, either in sports, occupational or clinical contexts. 

Considering our findings that perceived fatigability does not have an early relationship with performance in elderly people, it should be only be used with caution as a regulatory factor for intervention strategies, especially in the clinical context. It also seems necessary to carry out studies to identify consistent indicators that can be used in this and other populations to safely model intervention strategies to reduce or delay fatigue, improve functional and/or sports performance, and provide benefits for health and quality of life.

## 5. Conclusions

In conclusion, the results of this investigation show that for a sample of elderly participants, an upper limb isometric task produces changes in perceived and performance fatigability which are positively related in the final stage of the activity. Perceived fatigability evolved progressively with a major increase in the second half of the activity. Men performed the isometric activity longer than women but with a tendency to higher scores of perceived fatigability. 

Changes in perceived and performance fatigability are negatively related to BMI, especially in women, in whom age also affects perceived fatigability. Perceived and performance fatigability are also negatively correlated with health-related quality of life dimensions, with the social functioning dimension having a greater impact on perceived fatigability, and physical functioning and role functioning/physical dimensions on performance fatigability. 

## Figures and Tables

**Table 1:** Sample demographic and anthropometric characteristics.

Sample Characteristics	Total Sample Mean ± SD (Range)	Women Mean ± SD (Range)	Men Mean ± SD (Range)	*p*-Values	Effect Size Cohen’s d
Age (years)	72.50 ± 5.18 (65–85)	70.00 ± 1.00 (65–85)	73.00 ± 1.00 (65–82)	0.641 ^†^	3.000
Weight (kg)	73.34 ± 12.83 (48–100)	68.25 ± 12.25 (48–86.5)	78.44 ± 11.61 (57.90–100)	0.022 ^†^	0.854
Height (m)	1.59 ± 0.08 (1.48–1.76)	1.54 ± 0.50 (1.48–1.65)	1.65 ± 0.06 (1.57–1.76)	<0.001 ^†^	0,199
BMI (kg/m^2^)	28.72 ± 4.58 (21.56–37.94)	28.7 ± 1.28 (21.56–37.94)	28.74 ± 1.04 (23.42–35.61)	0.982 ^†^	0.034

In all analyses, *p* < 0.05 was considered statistically significant. ^†^ Independent samples Student’s *t*-test. Abbreviations: BMI, body mass index; SD, standard deviation.

**Table 2:** Sample SF36 health-related quality of life dimensions.

Health-Related Quality of Life Dimensions	Total Sample Mean ± SD (Range)	Women Mean ± SD (Range)	Men Mean ± SD (Range)	*p*-Value	Effect Size Cohen’s d
SF36 Physical functioning	74.38 ± 19.62 (30–100)	66.00 ± 5.00 (30–100)	83.00 ± 4.00 (40–100)	<0.001 ^†^	3.75
SF36 Role functioning/physical	80.47 ± 23.64 (31–100)	77.00 ± 6.00 (31–100)	84.00 ± 6.00 (31–100)	0.254 ^‡^	0.216
SF36 Pain	69.81 ± 25.32 (22–100)	59.00 ± 6.00 (22–100)	80.00 ± 6.00 (41–100)	0.023 ^‡^	0.40
SF36 General health	61.28 ± 19.82 (30–100)	55.00 ± 5.00 (30–92)	68.00 ± 5.00 (45–100)	0.051 ^†^	2.60
SF36 Vitality	51.88 ± 15.75 (25–80)	47.00 ± 4.00 (25–80)	57.00 ± 4.00 (35–80)	0.072 ^†^	2.50
SF36 Social functioning	88.67 ± 15.99 (38–100)	88.00 ± 4.00 (38–100)	89.00 ± 4.00 (50–100)	0.867 ^‡^	0.036
SF3 Role functioning/emotional	88.54 ± 18.54 (42–100)	82.00 ± 6.00 (42–100)	95.00 ± 2.00 (67–100)	0.270 ^‡^	0.215
SF36 Mental health	58.25 ± 17.97 (16–80)	51.00 ± 4.00 (16–80)	65.00 ± 4.00 (32–80)	0.039 ^‡^	0.37

In all analyses, *p* < 0.005 was considered statistically significant. ^†^ Independent samples Student’s *t*-test. ^‡^ Independent samples Mann-Whitney U test. Abbreviations: SF36, short-form health survey 36 item dimension; SD, standard deviation.

**Table 3:** Sample physical activity characterization.

Physical Activity	Total Sample Mean ± SD (Range)	Women Mean ± SD (Range)	Men Mean ± SD (Range)	*p*-Value	Effect Size Cohen’s d
Physical activity total time	646.53 ± 701.66 (0–2520)	668.00 ± 173.00 (0–2520)	625.00 ± 183.00 (0–2520)	0.669 ^‡^	0.078
Moderate physical activity time	479.44 ± 586.18 (0–2520)	696.00 ± 180.00 (0–2520)	263.00 ± 75.00 (0–840)	0.035 ^‡^	0.380
Vigorous physical activity time	234.03 ± 491.08 (0–2100)	107.00 ± 72.00 (0–840)	361.00 ± 155.00 (0–2100)	0.323 ^‡^	0.227

In all analyses, *p* < 0.05 was considered statistically significant. ^‡^ Independent samples Mann-Whitney U test. Abbreviations: SD, standard deviation.

**Table 4:** Perceived and performance fatigability variables.

Fatigability Variables	Total Sample Mean ± SD (Range)	Women Mean ± SD (Range)	Men Mean ± SD (Range)	*p*-Value	Effect Size Cohen’s d	Repeated Measures			
						*p*-Value	Effect Size Cohen’s d	*p*-Value	Effect Size Cohen’s d
PcFat 0%	0.47 ± 0.88	0.38 ± 0.89	0.56 ± 0.89	0.445 ^‡^	0.177	<0.001 ^Ξ^	0.76		
	(0.00–3.00)	(0.00–3.00)	(0.00–3.00)						
PcFat 25%	2.00 ± 1.48	1.69 ± 1.11	2.31 ± 1.75	0.237 ^†^	0.423			<0.001 ^§^	0.93
	(0.00–6.00)	(0.00–3.00)	(0.00–6.00)						
PcFat 50%	3.67 ± 2.00	3.03 ± 1.45	4.31 ± 2.29	0.069 ^†^	0.668	<0.001 ^Ξ^	0.88		
	(0.00–8.00)	(0.00–5.00)	(0.00–8.00)						
PcFat 75%	6.11 ± 2.18	5.5 ± 1.81	6.72 ± 2.41	0.094 ^‡^	0.299			<0.001 ^Ξ^	0.87
	(2.00–9.00)	(2.00–8.00)	(2.00–9.00)						
PcFat 100%	8.25 ± 2.29	7.75 ± 2.46	8.75 ± 2.05	0.210 ^‡^	0.243				
	(3.00–10.00)	(3.00–10.00)	(4.00–10.00)						
VPcFat 0–25%	1.53 ± 1.41	1.31 ± 0.98	1.75 ± 1.74	0.752 ^‡^	0.061	0.674	0.075		
	(0.00–5.00)	(0.00–3.00)	(0.00–5.00)						
VPcFat 25–50%	1.67 ± 1.18	1.34 ± 0.87	2.00 ± 1.38	0.118 ^†^	0.572			0.0054	0.5
	(0.00–4.00)	(0.00–3.50)	(0.00–4.00)						
VPcFat 50–75%	2.44 ± 1.16	2.47 ± 1.22	2.41 ± 1.13	0.876 ^‡^	0.034	0.154	0.713		
	(0.50–4.50)	0.50–4.00)	(1.00–4.50)						
VPcFat 75–100%	2.14 ± 1.34	2.25 ± 1.34	2.03 ± 1.37	0.669 ^‡^	0.079				
	(0.00–6.00)	(0.50–5.50)	(0.00–6.00)						
VPcFat 0–50%	3.20 ± 2.12	2.66 ± 1.41	3.75 ± 2.59	0.151 ^†^	0.521	0.0113	0.66		
	(0.00–8.00)	(0.00–5.00)	(0.00–8.00)						
VPcFat 50–100%	4.58 ± 1.97	4.72 ± 2.23	4.44 ± 1.74	0.694 ^†^	0.140				
	(1.00–9.50)	(1.00–9.50)	(1.00–7.00)						
VPcFat 25–75%	4.11 ± 1.86	3.81 ± 1.64	4.41 ± 2.07	0.375 ^†^	0.321				
	(1.00–8.00)	(1.50–6.50)	(1.00–8.00)						
TTF (sec)	472.25 ± 296.79	371.63 ± 221.18	572.88 ± 333.94	0.043 ^‡^	0.360				
	(128–1443)	(128–1012)	(286–1443)						

In all analyses, *p* < 0.05 was considered statistically significant. ^†^ Independent samples Student’s *t*-test; ^‡^ Independent samples Mann-Whitney U test; ^§^ Paired samples Student’s *t*-test; ^Ξ^ Wilcoxon signed-rank test. Abbreviations: PcFat, Perceived fatigability; TTF, time to task failure; SD, standard deviation.

**Table 5:** Relations between perceived and performance fatigability.

Time to Task Failure (TTF)			
	Total Sample	Women	Men
Perceived fatigability (PcFat)	PcFat 75%	PcFat 75%	PcFat 25%
	(ρ = 0.544 **, 95% CI [0.22, 0.76]	(ρ = 0.615 *, 95% CI [0.12, 0.86])	(ρ = 0.568 *, 95% CI [0.06, 0.84])
	PcFat 100%	PcFat 100%	PcFat 75%
	(ρ = 0.645 **, 95% CI [0.35, 0.82])	(ρ = 0.848 **, 95% CI [0.55, 0.95])	(ρ = 0.522 *, 95% CI [0.00, 0.82])

* Statistically significant correlation *p* < 0.05; ** Statistically significant correlation *p* < 0.01 (2 tail). Abbreviations: PcFat, Perceived fatigability.

**Table 6:** Correlation between perceived fatigability variables and sample characteristics.

Perceived Fatigability (PcFat)			
	Total Sample	Women	Men
Sample characteristics		Age-PcFat 25%	
		(r = 0.660 **, 95% CI [0.24, 0.87])	Vigorous-PcFat 100%
	Vigorous - PcFat 75%	Age-PcFat 50%	(ρ = 0.560 *, 95% CI [0.05, 0.84])
	(ρ = 0.363 *, 95% CI [0.00, 0.64])	(r = 0.524 *, 95% CI [0.038, 0.81])	
		BMI - PcFat 100%	SF36 Pain - PcFat 50%
		(ρ = −0.507 *, 95% CI [−0.81, 0.02])	(ρ = −0.543 *, 95% CI [−0.83, −0.03])
	SF36 Social functioning - PcFat 75%		SF36 Pain - PcFat 75%
	(ρ = −0.411 *, 95% CI [0.06, 0.67])	SF36 Vitality - PcFat 25%	(ρ = −0.556 *, 95% CI [−0.84, −0.04]))
		(r = −0.559 *, 95% CI [−0.83, −0.09])	SF36 Vitality - PcFat 50%
			(r = −0.533 *, 95% CI [−0.81, −0.05])

* Statistically significant correlation *p* < 0.05; ** Statistically significant correlation *p* < 0.01 (2 tail). Abbreviations: BMI, body mass index; SF36, short-form health survey 36 item dimension.

**Table 7:** Correlation between performance fatigability variables and sample characteristics.

Time to Task Failure (TTF)			
	Total Sample	Women	Men
Sample characteristics	BMI (ρ = −0.471 **, 95% CI [−0.71, −0.13]) SF36 Physical functioning (ρ = 0.471 **, 95% CI [0.13, 0.71]) SF36 Role functioning/physical (ρ = 0.381 *, 95% CI [0.02, 0.65])	BMI (ρ = −0.629 **, 95% CI −0.87, −0.14]) SF36 Physical functioning (ρ = 0.506 *, 95% CI [−0.02, 0.81])	No significative correlation

* Statistically significant correlation *p* < 0.05; ** Statistically significant correlation *p* < 0.01 (2 tail). Abbreviations: BMI, body mass index; SF36, short-form health survey 36 item dimension.

## Data Availability

The dataset supporting the conclusions of this article is available upon request to h.smigueis@udc.es in the Research, Health and Podiatry Group, Department of Health Sciences, Faculty of Nursing and Podiatry.
